# Study of the Absorption of Electromagnetic Radiation by 3D, Vacuum-Packaged, Nano-Machined CMOS Transistors for Uncooled IR Sensing

**DOI:** 10.3390/mi12050563

**Published:** 2021-05-16

**Authors:** Gil Cherniak, Moshe Avraham, Sharon Bar-Lev, Gady Golan, Yael Nemirovsky

**Affiliations:** 1 Electrical Engineering Department, Technion—Israel Institute of Technology, Haifa 32000, Israel; gilcher@campus.technion.ac.il (G.C.); smoa@technion.ac.il (M.A.); sharonb@technion.ac.il (S.B.-L.); 2 Department of Electrical Engineering and Electronics, Ariel University, Ariel 40700, Israel; gadygolan@gmail.com

**Keywords:** thermal sensors, TMOS sensor, finite difference time domain, optical and electromagnetics simulations

## Abstract

There is an ongoing effort to fabricate miniature, low-cost, and sensitive thermal sensors for domestic and industrial uses. This paper presents a miniature thermal sensor (dubbed TMOS) that is fabricated in advanced CMOS FABs, where the micromachined CMOS-SOI transistor, implemented with a 130-nm technology node, acts as a sensing element. This study puts emphasis on the study of electromagnetic absorption via the vacuum-packaged TMOS and how to optimize it. The regular CMOS transistor is transformed to a high-performance sensor by the micro- or nano-machining process that releases it from the silicon substrate by wafer-level processing and vacuum packaging. Since the TMOS is processed in a CMOS-SOI FAB and is comprised of multiple thin layers that follow strict FAB design rules, the absorbed electromagnetic radiation cannot be modeled accurately and a simulation tool is required. This paper presents modeling and simulations based on the LUMERICAL software package of the vacuum-packaged TMOS. A very high absorption coefficient may be achieved by understanding the physics, as well as the role of each layer.

## 1. Introduction

There has been a great deal of interest in low-cost uncooled IR sensors in recent years, which may bolster a wide range of new applications, such as consumer electronics, smart homes, Internet of Things (IoT) devices, and mobile applications. Over the years, thermal sensors have been extensively applied to uncooled passive infrared (PIR) sensing. Thermal detection sensors are based on mechanisms that change some measurable property of a material due to the temperature rise of that material as caused by the absorption of electromagnetic radiation. Of these, the most important state-of-the-art thermal detectors are microbolometers, thermopiles, and pyroelectric IR (PIR) sensors [1,2,3,4,5,6].

Commercially available PIR sensors are usually based on decades-old pyroelectric detector technology. The main drawback of current pyroelectric PIR detectors is that the sensors can only detect moving objects and not the presence of hot objects. Furthermore, as the response times of these sensors are relatively high, fast moving targets are often not detected. They can also fail to detect intruders that move slowly or crawl. They also suffer from false events, which are particularly common at elevated temperatures.

Micromachining has been the enabling technology for sensitive thermal sensors, which require very low thermal mass and very low thermal conductivity [7]. When an optical power irradiates a micro-machined thermal sensor packaged in vacuum, its steady state temperature increases by ΔTss = ηPir/Gth, where Gth [W/K] is the thermal conductance of the holding arms and η is the absorbing efficiency of the radiation. 

The advent of microelectromechanical systems (MEMS) and nanoelectromechanical systems (NEMS) technologies in CMOS technology has enabled the production of high performance bolometers and thermopiles. CMOS and its derivative CMOS-SOI are the prevalent microelectronics technologies and the key to a significant cost reduction in many monolithically integrated electro-optical sensors.

Microbolometers are still relatively expensive as they require additional fabrication steps, such as vanadium oxide deposition, on top of standard surface micromachining processes. Thermopiles are being compatible with standard CMOS processes that allow low-cost production with large volumes but require powerful amplifiers since the internal signal is low.

Recently, novel uncooled thermal sensors based on CMOS-SOI technology have been extensively pursued [8,9,10,11,12,13,14], mainly for IR and THz detection, and more recently for gas sensing [15,16,17,18]. The sensor, dubbed TMOS, is based on a suspended micro- or nano-machined transistor fabricated in standard CMOS-SOI process and released by dry etching. The thermally isolated transistor, operating at subthreshold, converts small temperature changes to electrical signals as the transistor I-V characteristics are strongly dependent upon temperature at a subthreshold level [12].

At present, the most advanced TMOS processing is based on a nanometric 0.13-µm CMOS SOI technology and is implemented with 8-inch wafers. The silicon technology is available for advanced yet standard CMOS and MEMS FABs [19], offering wafer-level processing and packaging (WLP) with integrated optical windows and filters above the vacuum. As a result, there is a considerable and exceptional potential for cost reduction. Since the TMOS may be operated at a subthreshold level, thus consuming very low power, it may be powered by a battery, enabling a wide range of applications related to mobile phones, smart homes, security, and IoT. This low power feature of the TMOS is a great advantage in comparison to the passive bolometers for example, which currently dominate the IR imaging market. In addition, since the TMOS is a transistor, which is an active device exhibiting large internal gain even at subthreshold, it exhibits unprecedented temperature sensitivity TCV[K^−1^] = (dV/dT)/V and very high responsivity in terms of voltage/wattage in comparison to the commercial passive thermal sensors such as pyroelectric sensors, thermopiles, and diodes.

This paper presents modeling and simulations based on the LUMERICAL software package [20] for the absorption of IR radiation by a vacuum-packaged TMOS sensor. Since the TMOS is processed in a CMOS-SOI FAB and is comprised of multiple thin layers, the absorbed electromagnetic radiation cannot be modeled accurately and a simulation tool is required. Section 2 discusses the choice of the simulation tool and the advantages and drawbacks of LUMERICAL. Section 3 presents the design of a TMOS pixel, as well as the vacuum-packaged TMOS. Incident IR radiation is first transmitted through the upper silicon wafer, which provides the optical window. The transmitted electromagnetic radiation is absorbed in the MEMS/NEMS released TMOS. Section 4 discusses the absorption, reflection, and transmission of the front optical window cap. Since the optical window has a simple structure, the simulation is compared with analytical numerical modeling with MATLAB. The correspondence between the simulation, modeling and measurements validates the results. Section 5 is the heart of this paper and presents the optimized absorption of the TMOS with an impedance matching layer made of TiN. Section 6 summarizes the paper. The goal of this study is to give physical insight regarding the layers and mechanisms that play primary roles in TMOS electromagnetic absorption, as well as to assist in the use of LUMERICAL for simulations.

## 2. The LUMERICAL Simulation Tool

The absorption simulation was carried out using the LUMERICAL finite-difference time-domain (FDTD) tool and the LUMERICAL knowledge base [20]. It is a state-of-the-art tool for solving Maxwell’s equations in complex geometries, which allows solving and analyzing electromagnetics in complex photonics problems. FDTD splits the simulated region into many mesh cells and solves the equations relating to the time and space dependence of the electromagnetic fields at the cell boundaries. Useful quantities can be calculated by using these data, such as the Poynting vector and the transmission/reflection of radiation or absorbed power.

One of the main advantages of LUMERICAL FDTD is its ability to simulate very thin layers that are relative to the radiation wavelength and to use a precise material refractive index that is frequency-dependent in order to get an accurate analysis of the model. Furthermore, LUMERICAL software allows creation of time-domain field propagation simulations that contribute to the understanding of the nature of electromagnetic absorption in the studied system.

There are also several challenges associated with this software:

• The division of the space into mesh points require that a unit cell will be much smaller than the shortest wavelength and smaller than the smallest geometrical feature. This might require multiple mesh points and result in very long simulation durations;
• Introducing boundary conditions at the boundary of the mesh can result in the introduction of errors;
• Resonators with high Q factor require very long simulation times to converge, if at all.

### A Brief Comparison between FEM and FDTD

Electromagnetic simulators solve Maxwell equations, which correspond to the initial conditions and boundary conditions. Due to the wide variety of types, shapes, and dimensions of problems, there is no single simulation solver method that is best suitable for all problems and applications. For 3D problems, there are two common solver methods: FDTD, which is used in this paper with LUMERICAL, and finite element methods (FEMs), which are used in many commercial solvers like HFSS and COMSOL Multiphysics.

The simulation processes for the FDTD and FEM solvers are similar. The first step is to define the physical model, which includes the geometry and the properties of the materials. The second step is to set up the simulation, which includes defining the simulation’s general settings, boundary conditions and discretizing the physical model to cells. The last step is to run the simulation and postprocess the results.

Although the simulation processes are similar for both methods, the implementation difference between the methods in the way they each solve and discretize the domain may have a huge effect on the results and computation time for different applications.

In the FDTD approach, the region of the simulation is defined. The simulation domain is discretized by a rectangular Cartesian style mesh cell. For each cell, the FDTD method solves Maxwell equations on each cell and time step. The FDTD method solves the equation in the time domain, which makes it usually more suitable for time domain reflectometry. Another advantage for solving the equation in the time domain is that by one simulation the results for broadband frequencies can be achieved.

In the FEM method, the domain volume is discretized to a finite number of elements and nodes, usually by tetrahedron cell mesh. The field is approximated for each cell. Among the nodes, a piecewise polynomial solution is assumed and applying the boundary conditions and simulation properties yields a sparse matrix to determine the fields. Unlike the FDTD method, the FEM method solves the equations in the frequency domain, which makes it more suitable for example for resonators or other high-Q circuits. RF engineers and researchers prefer to use CST or HFSS commercial software while electro-optical scientists may appreciate the advantages of LUMERICAL.

## 3. TMOS Pixel Design

**Figure 1:**
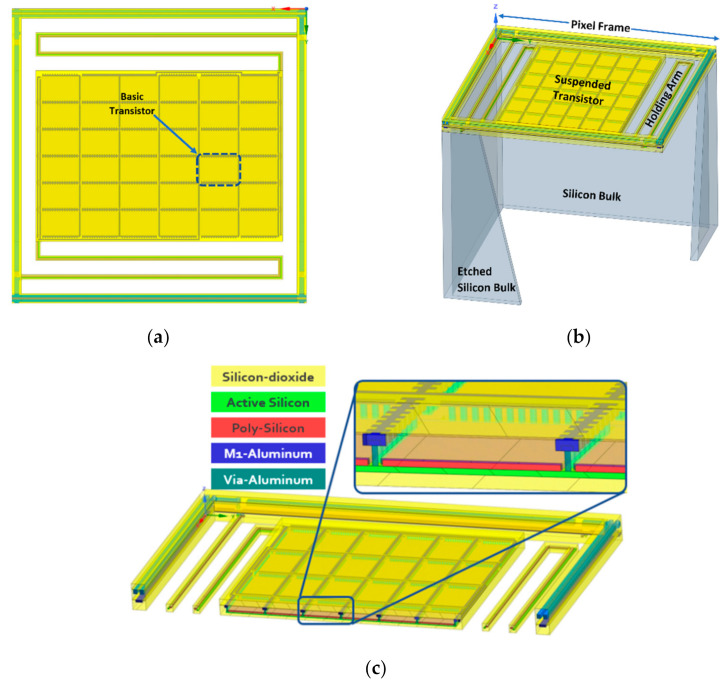
TMOS single nano-machined pixel. (**a**) Overview; (**b**) 3D model; (**c**) cross section of the pixel.

Figure 1 presents a single nano-machined pixel, including an overview of the layout and a 3D model. The layout of a typical pixel is shown in Figure 1a. The suspended TMOS is released by RIE and DRIE dry etching. For reproducible processing across 8-inch wafers, all gaps must be the same.

The TMOS released transistor of the pixel has the designed form factor W/L, where W is the width and L is the length of the transistor. It is based on a serial and parallel combination of the largest transistor that the PDK provides in order to be able to use PDK models. For example, large channel length, L is obtained by serially connecting two transistors and the required W is obtained by combining in parallel several transistors.

**Figure 2:**
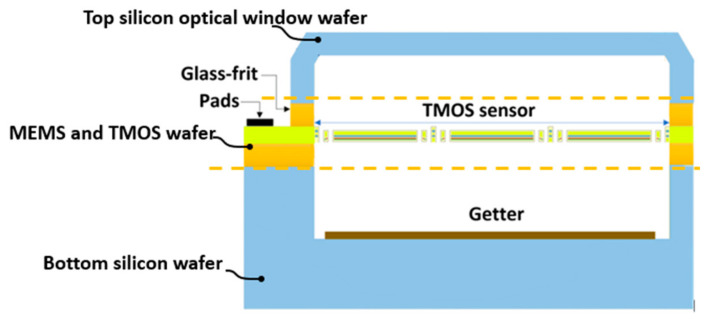
Schematic of the wafer-level package architecture with a getter layer for a high vacuum.

The vacuum-packaged device is shown in Figure 2 [21].

The challenges for wafer-level vacuum packages with MEMS devices are well established and reported in the literature [22]. In the case of optical MEMS sensors, such as thermal sensors, the package plays an important role in the performance, as discussed below.

## 4. The Absorption, Reflection, and Transmission of the Top Silicon Optical Window

### 4.1. Modeling

The TMOS cap is fabricated by two layers: a silicon wafer of the order of 100 µm and a much thinner silicon dioxide layer. The optical wave transmission of this Fabry–Perot cavity can be analytically calculated with transfer matrices of the electric fields [23].

**Figure 3:**
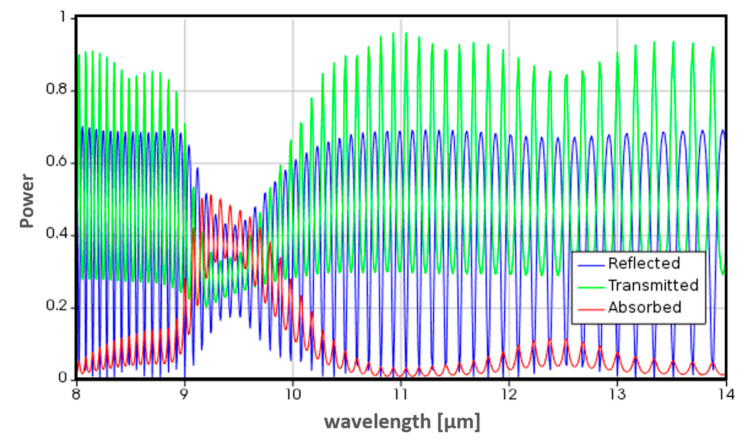
The modeled absorption, reflection, and transmission of the optical window as a function of the wavelength. The silicon wafer size is ~100 µm and the oxide layer is of the order of 0.1 µm.

Figure 3 exhibits the absorption, reflection, and transmission of the top optical window.

### 4.2. LUMERICAL Simulation

**Figure 4:**
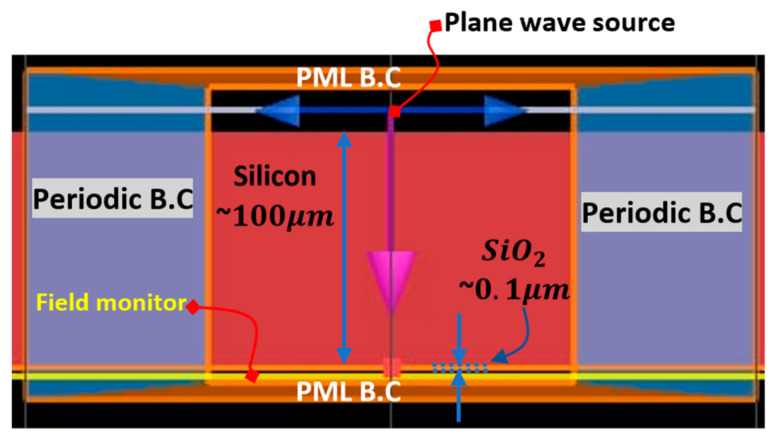
The simulation model and setup. The simulated area is outlined by the orange rectangle. The lateral dimensions are in the order of 0.1 µm. PML B.C.: perfectly matched layer boundary conditions.

A FDTD model has been created in LUMERICAL as described in Figure 4 in order to simulate the TMOS optical window.

As can be seen in Figure 4, on the sides of the FDTD region periodic boundary conditions were applied and on the top and bottom of the FDTD region a perfectly matched layer (PML) boundary conditions were applied. A radiation source with the wavelength range of 5–20 μm was set above the TMOS cap. The refractive indices of the materials were set by the software using Palik data [20,24].

**Figure 5:**
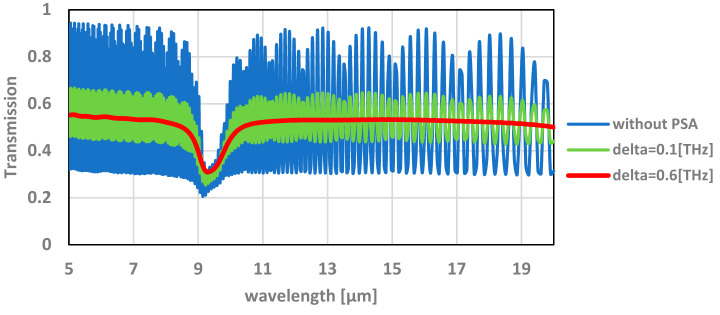
Simulations results for the transmission of the TMOS optical window as a function of the wavelength. PSA: partial spectral averaging (δ: delta, see Section 4.3).

The simulation results are shown in Figure 5.

**Figure 6:**
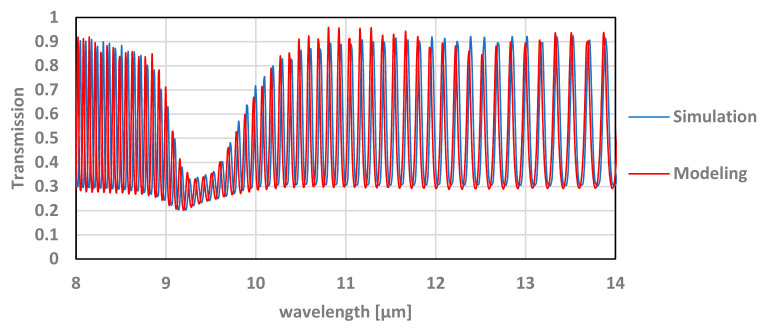
Simulation and modeling of transmission results as a function of the wavelength.

The simulations and modeling show good agreement, as can be seen in Figure 6.

**Figure 7:**
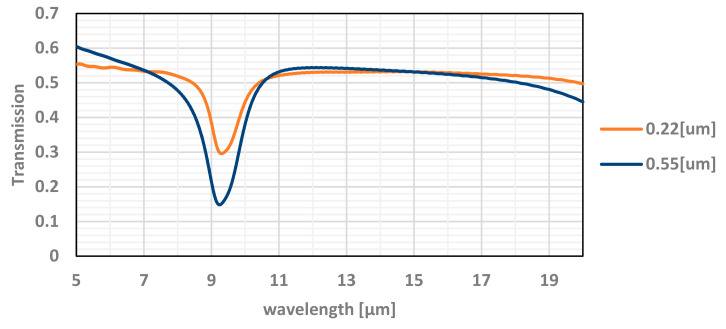
The simulated optical transmittance of the TMOS optical window for different silicon dioxide thickness as a function of the wavelength. The simulated results are in good agreement with measured results.

The measured transmission results, compared with the simulation, as shown in Figure 7, are very different and do not show the resonance lines. A good fit between measurements and simulation or modeling requires partial spectral averaging, and δ (delta) estimation, as explained below.

### 4.3. Comparison with Measurements

The optical window that was simulated was a Fabry–Perot cavity with two dielectric slab devices. One of the slabs was much wider (>100 µm) than the typical wavelength used for the measurements. Therefore, inside this slab, we expected to find a standing wave. During measurement, the transmitted power through the device was measured for a range of wavelengths. The simulation calculates the transmission for a specific single wavelength, whereas measurement is experimentally conducted around a certain bandwidth, since it is difficult to produce a monochromatic source. Therefore, the calculated simulation should be averaged by a convolution with a Lorentzian weighting function, which is a function mainly used to characterize narrow spectrum lines, such as emission by atoms, laser radiation, etc. [20]. 

The Lorentzian weighting function is defined by:

(1) $${\left|h\left(\omega ,\omega^{\prime }\right)\right|}^{2}=\frac{\delta }{\left(\omega -\omega^{\prime }\right)+{\left(\pi \delta \right)}^{2}}$$

where *δ* is the FWHM (full width at half maximum) and *ω* is the Lorentzian center frequency. The value of *δ* can be evaluated by differentiating the relation λ = c/f with respect to f, resulting in |df| = f/λ dλ, where dλ is the measurement error of the wavelength. The value of df can be used for the evaluation of the FWHM. As described in Figure 5, the value of the FWHM used in the simulation was *δ* = 0.6 (THz), which corresponds to a value of dλ = 200 (nm). This value yields a match between the measurements and simulation when using that value.

## 5. Optimized Absorption of the TMOS Sensor with an Impedance Matching Layer with the Right Thickness and Location

To gain an understanding of the electromagnetic absorption of the TMOS sensor, the classical propagation of waves should be applied. The dielectric layers of the TMOS released transistor (see Figure 1c) are comprised of silicon dioxide and silicon nitride. The silicon oxide bond absorbs at ~9.3 µm, while the silicon nitride bond absorbs at ~11.5–12 µm [25]. The physical description becomes complicated since there are several built-in Fabry–Perot resonators and the LUMERICAL simulations become very useful here. Furthermore, it is well established from bolometers that an impedance matching layer with the correct thickness and placement allows optical absorption with 90% efficiency along large arrays. The concept of impedance matching layer for an ideal λ/4 optical cavity is described in Appendix A [26]. The transmission line description, discussed in Appendix B, provides an intuitive description of an ideal optical cavity.

It is apparent that in order to reduce reflection and enhance absorption, an impedance matching layer is mandatory. This layer should provide the required impedance at the frequency of the incident radiation. It should be based on a standard metallization available in the CMOS FAB. Luckily, Ti and TiN may provide this layer. At DC, the specific resistivity of these layers is too low, but at the IR frequency of interest (around 10^13^ Hz) the values increase by a factor of ~3 because of the plasma effect [25].

**Figure 8:**
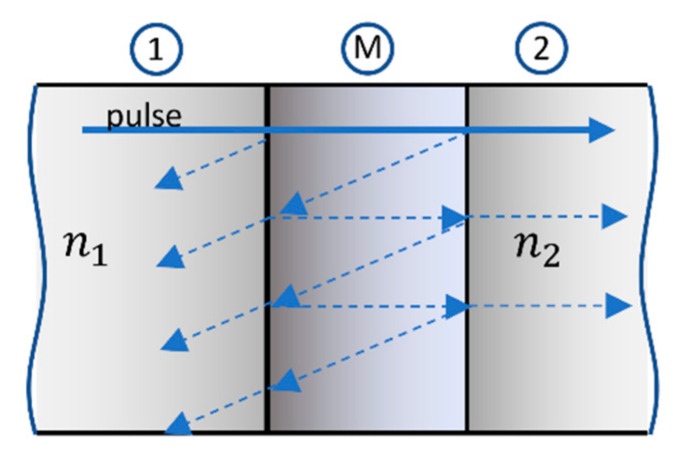
Schematic of an optical system consisting of an optical material (1) with n_1_, thin metal layer (M), and optical material (2) with n_2_.

In the TMOS sensor case, the modeling is more complicated than that of Appendix B. We assume that a thin “impedance matching layer” is sandwiched between two optical materials (the interlevel dielectric material of the TMOS). When the skin depth into the metal is larger than its thickness, the metallic film can be considered as a regular optical layer (that may be described by the Drude model [25]). In this case, the Salisbury screen becomes a dielectrically-coated Salisbury sheet (DSS) [26,27,28,29] (see an example in Figure 8):

As discussed above, the definition of “impedance matching layer” in this case is much more complicated: What should be the thickness and the location of this layer in the cross section of Figure 1c? Theoretically, Fabry–Perot or transmission line modeling may be performed, but LUMERICAL simulations are very effective and useful here.

At this point, it is important to remind the reader that the accuracy of the LUMERICAL simulation is very much dependent upon the mesh dimensions. Accuracy increases if the mesh is less than one tenth of the wavelength. Furthermore, a simple “sanity check” should be performed to validate the results. The sum of absorption, reflection, and transmission should be 1. If it is higher than 1, the mesh should be redefined and be reduced in dimensions.

We present simulation results below that demonstrate how the thickness of the layer (Section 5.1) and location (Section 5.2) affect absorption. At certain bandpass regions, high absorption of 90% may be obtain (Section 5.3).

### 5.1. The Effect of the Thickness of the TiN

**Figure 9:**
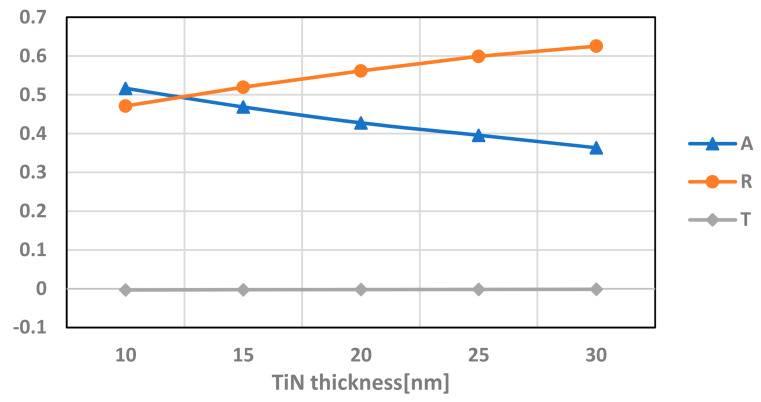
Absorption (A), reflection (R), and transmission (T) of the transistor with varying thicknesses of the TiN layer. Simulation is performed at 9.5 µm wavelength.

To begin with, we wanted to examine the influence of the thickness of the TiN layer on the absorbance. We simulated the model for varying thickness from 10 nm up to 30 nm, all positioned in the same height of 2.16 µm from the bottom of the Box. The absorption, reflection, and transmission of this simulation can be seen in Figure 9.

It can be seen from Figure 9 that the absorption in the transistor is more significant as the layer thickness decreases and can be improved from 36% up to 52%.

### 5.2. The Effect of the Location of the TiN along the Upper Silicon Oxide

**Figure 10:**
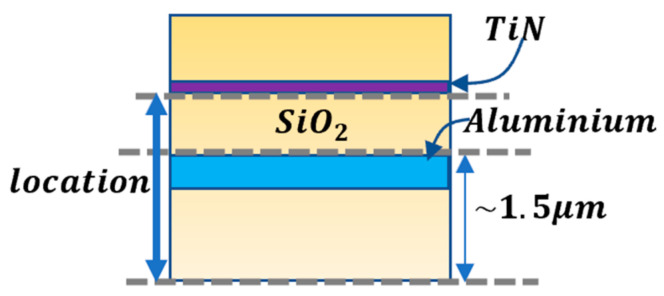
Schematic of the TiN location in the TMOS sensor.

After optimizing the thickness of the TiN layer, we continued with optimizing the absorbance of the sensor by changing the location of the TiN along the upper silicon oxide, which starts after the last metal layer at the height in the order of ~1.5 µm (see Figure 10).

The absorption, reflection, and transmission were simulated for different locations along the silicone oxide for 10-nm and 20-nm layers of TiN.

**Figure 11:**
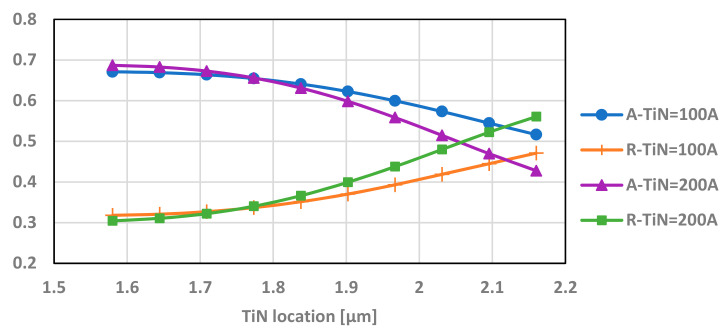
Absorption (A), reflection (R), and transmission (T) of the transistor with varying locations of the TiN layer along the upper silicon oxide for 10-nm and 20-nm layer thickness. This simulation is performed at a 9.5-µm wavelength.

It can be seen in Figure 11 that the absorbance is higher at a lower TiN placement. As shown in Section 5.1, the higher absorption is reached by the 10–20 nm TiN layer and is slightly higher for 20 nm layer of TiN.

### 5.3. Simulation of Absorption at an Optical Bandpass

**Figure 12:**
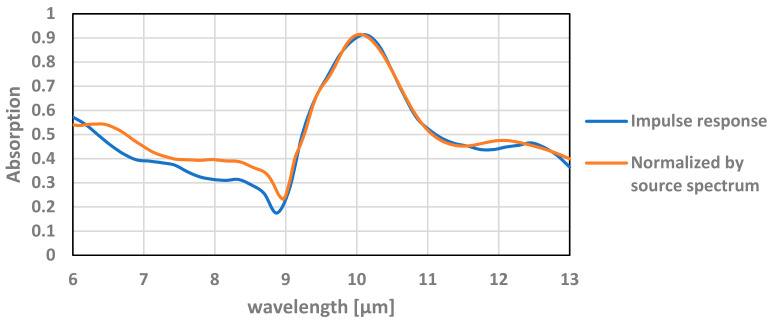
Absorption of the TMOS transistor with a TiN layer thickness of 20 nm located at 1.58 µm (see Figure 10) as a function of the wavelength.

In practice, the TMOS sensor is provided with a bandpass filter that is determined by the use case. We present the simulation below within a bandpass of 6–13 µm. The simulations were performed with default continuous wave normalization, where in this state the power monitors are normalized by the Fourier transform of the source pulse. In other words, it returns the impulse response of the system. For most applications this is the best choice, but in some applications there may be critical mismatch. For this paper, there is a good agreement between the field’s results yield by the normalized power over the source spectrum to the impulse response of single frequency simulations, as can be seen in Figure 12.

**Figure 13:**
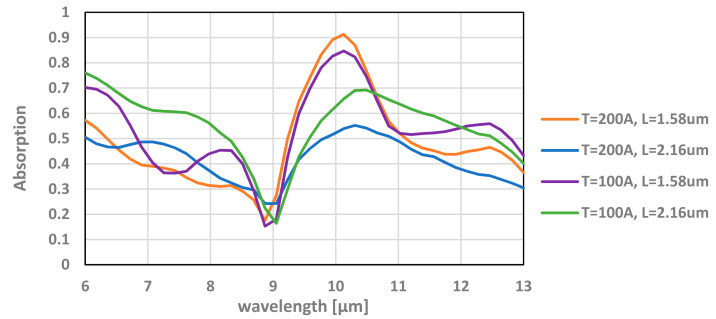
Absorption of the TMOS transistor for various thicknesses (T) and locations (L) as a function of the wavelength.

For the optimized thickness and location of the TiN layer, an absorption of about 90% can be achieved. The simulation results for various TiN layer thickness and location can be seen in Figure 13.

**Figure 14:**
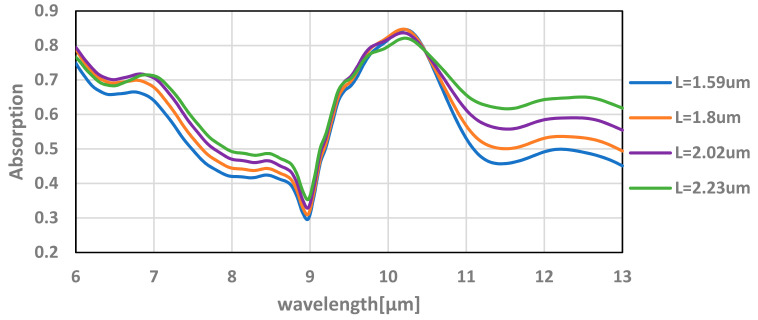
Absorption of the TMOS transistor with additional SiN layer with a thickness of 0.1 µm for various locations (L) as a function of the wavelength.

Figure 14 shows the simulation results for additional SiN layer with thickness of 0.1 µm for various locations. Though the maximum absorption decreases by about 10% around 10 µm for most of the bandpass spectrum, the absorption increases by about 20%.

## 6. Summary

The goal of this study was to provide physical insight and an intuitive approach to the layers and mechanisms that play primary roles regarding 3D, vacuum-packaged, nano-machined TMOS electromagnetic absorption.

The main parameters that determine the overall absorption efficiency of the vacuum-packaged TMOS sensor are (i) the *transmission* of the optical window and (ii) the *absorption* of the released TMOS sensor, which should be as high as possible. The latter is significantly increased by the impedance matching layer (a dielectric-coated Salisbury sheet) [27,28,29].

**Figure 15:**
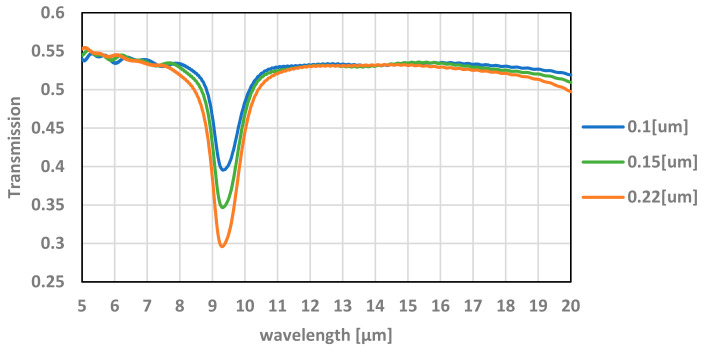
The effect of the thickness of the oxide on the transmission of the optical window. The simulation is achieved with PSA averaging and δ = 0.6 (THz).

As can be seen in Figure 15, the degrading effect of the oxide, which is part of the optical window, upon the transmission of the electromagnetic radiation in the bandpass of interest (>5 µm) is clearly observed. The average transmission is ~55% while at the resonance wavelengths (around 9.3 µm) it is significantly reduced. By integrating an antireflection (AR) coating on the optical window, its transmission may be significantly increased.

The absorption of the TMOS sensor may be optimized with the LUMERICAL simulations. By optimizing the thickness of the *impedance matching layer* of a thin TiN layer and its location along the upper inter-level dielectric of the TMOS, a significant absorption of 90% may be achieved. Adding more than one impedance matching layer may also enhance absorption [27] (chapter 9). Physical insight may be gained by applying transmission lines modeling and Smith chart modeling, but this is beyond the scope of this paper [27,28,29].

**Figure 16:**
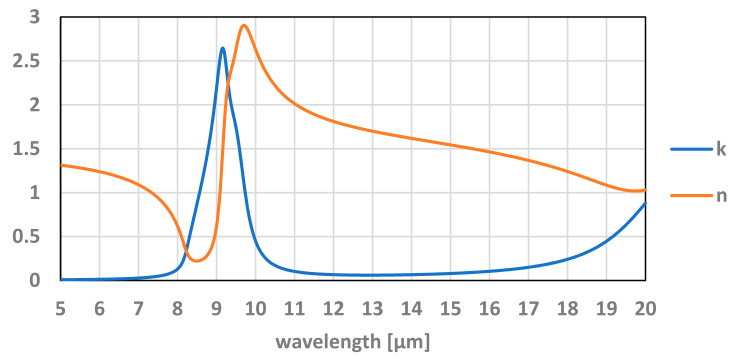
The refractive index of SiO_2_ as a function of the TMOS bandpass wavelength where (**n**) is the real part and (**k**) is the imaginary part.

Moreover, the properties of thin layers are different from the bulk materials and depend on the deposition technology. Appendix C reports the values used in this study. Better simulations may be achieved if the actual TMOS main dielectric layers and refractive index are measured. The values of n(λ) and k(λ) play a role in determining the exact values of absorption. For example, the refractive index for the main TMOS material SiO_2_ used in the simulation can be seen in Figure 16.

In summary, the LUMERICAL software package is an excellent tool; however, like all software, it requires understanding of the limitations of the tool and the underlying approximation of results. A designer that is also an expert in transmission line theory and Smith charts may achieve very high absorption of the order of 90% within the bandpass of interest.

## Figures and Tables

**Figure A1:**
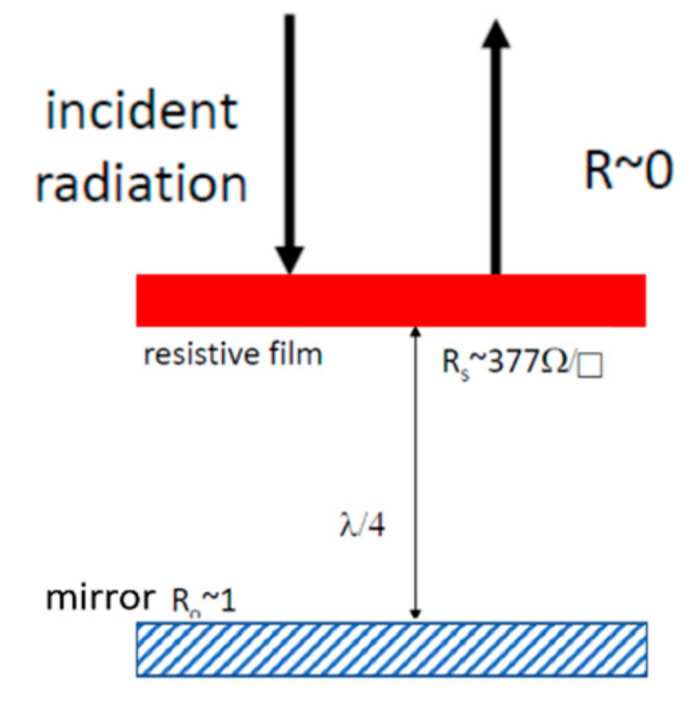
Ideal optical cavity based on Salisbury screen.

**Figure A2:**
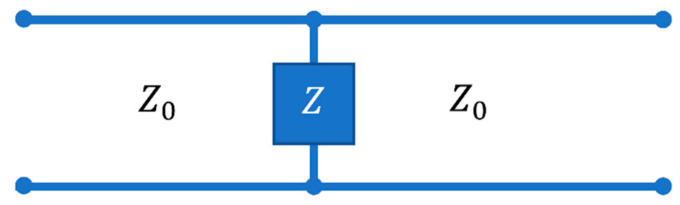
Schematic model of transmission line with pixel with impedance Z. (note Z_0_ is the impedance of the air or vacuum).

**Figure A3:**
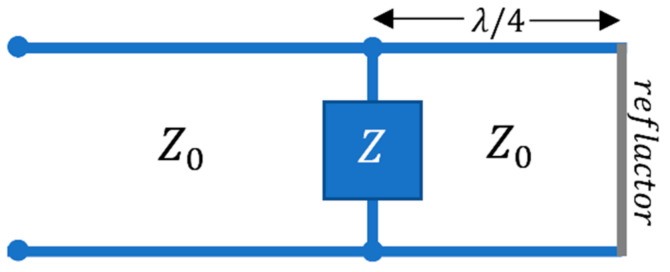
Schematic model of transmission line with pixel with impedance *Z* and λ/4 transformer for perfect matching.

## Appendix A. Impedance Matching Layer: Wave Absorption with a Salisbury Sheet

It is well established that if a sheet with a thin layer of 377 Ω/□ (ohm per square), known as a Salisbury sheet [26], is placed at λ/4 in front of a perfectly conducting sheet, the incident wave in air will be completely absorbed in the sheet and dissipated as heat, without any reflection, as shown in Figure A1.

The 377 Ω/□ (ohm per square) is the intrinsic impedance of air or vacuum and the sheet of resistive film (the Salisbury Sheet) is known as “impedance matching layer”.

In this arrangement, the impedance presented to the incident wave at the sheet is the impedance of the sheet backed by an infinite impedance, since at λ/4 the perfectly conducting layer at the bottom is transformed to an infinite impedance. As a result, the incident wave is totally absorbed; however, there is a standing wave and energy circulation between the resistive and conducting sheets and this energy is dissipated as heat in the resistive sheet.

## Appendix B. Modeling *the Ideal Optical Cavity* with Transmission Lines

The easiest way to model the ideal optical cavity is by using transmission line theory. Optical waves travel through space as through an infinite transmission line. A pixel with an electrical impedance Z can be modeled as a parallel load between two halves of this line (see Figure A2). The refection coefficient is given by:

(A1) $$\Gamma =\frac{Z_{0}-(Z||Z_{0})}{Z_{0}+(Z||Z_{0})}$$

For *Z* = *Z*_0_ the reflection is 1/3.

To maximize absorption, we need to minimize reflection. This is achieved with λ/4 optical cavity. We can achieve a perfect matching by using a “λ/4 transformer” terminated by a perfect conductor (*Z*_L_ = 0), which transforms the impedance seen by the waves to an infinite impedance, in parallel to the impedance of the pixel (see Figure A3).

Accordingly, the reflection coefficient is:

(A2) $$\Gamma =\frac{Z_{0}-(Z||Z_{0})}{Z_{0}+(Z||Z_{0})}=\frac{Z_{0}-(Z||\infty )}{Z_{0}+(Z||\infty )}=\frac{Z_{0}-Z}{Z_{0}+Z}$$

Hence, if *Z* = *Z*_0_, the reflection is canceled. Since both, the optical cavity and the reflector, do not absorb energy, all the energy is absorbed by the pixel—the only dissipative element.

## Appendix C. Optical and Material Parameters of the Main Layers Used in the Simulations

**Table A1:** Refractive index of TiN, SiN, and SiO_2_ at λ = 9.5 µm.

Material	N	K
TiN	8.5	16.4
SiN	1.56	0.71
SiO_2_	2.7	1.71

Note: It is well known that the physical properties of thin layers are different from the properties of bulk materials and depend on the deposition technology.
